# Cleaning Paintings

**Published:** 1871-10

**Authors:** 


### CLEANING PAINTINGS,

Or other oiled surfaces, as the walls of a room, is best done thus: Dip a piece of very soft flannel in warm water, squeeze it as dry as possible, dip it in very finely-powdered French chalk, rub gently, then wash with a fine, clean sponge in water, and wipe dry with wash-leather. A grease spot on a wall or painting is so unsightly, it is worth while for every housekeeper to bear in mind the best method of removal.

